# Survey of the effects of internet usage on the happiness of Japanese university students

**DOI:** 10.1186/s12955-019-1227-5

**Published:** 2019-10-11

**Authors:** Momoko Kitazawa, Michitaka Yoshimura, Hidefumi Hitokoto, Yuka Sato-Fujimoto, Mayu Murata, Kazuno Negishi, Masaru Mimura, Kazuo Tsubota, Taishiro Kishimoto

**Affiliations:** 1 Department of Neuropsychiatry, Keio University School of Medicine, 35 Shinanomachi, Shinjuku, Tokyo, 160-8582 Japan; 2 Department of Sleep-Wake Disorders, National Institute of Mental Health, National Center of Neurology and Psychiatry, Kodaira, Tokyo, Japan; 3 Department of Culture, Faculty of Humanities, Fukuoka University, Jonan, Fukuoka, Japan; 4 Faculty of Health Sciences, Gumma Paz University, Takasaki, Gumma Japan; 5 Department of Student Affairs, Mie University, Tsu, Mie Japan; 6 Department of Ophthalmology, Keio University School of Medicine, Shinjuku, Tokyo, Japan

**Keywords:** Happiness, Well-being, Social networking service, School performance, Internet dependence, Sleep, Young adults

## Abstract

**Background:**

Besides research on psychiatric diseases related to problematic Internet use (PIU), a growing number of studies focus on the impact of Internet on subjective well-being (SWB). However, in previous studies on the relationship between PIU and SWB, there is little data for Japanese people specifically, and there is a lack of consideration for differences in perception of happiness due to cultural differences. Therefore, we aimed to clarify how happiness is interdependent on PIU measures, with a focus on how the concept of happiness is interpreted among Japanese people, and specifically among Japanese university students.

**Methods:**

A paper-based survey was conducted with 1258 Japanese university students. Respondents were asked to fill out self-report scales regarding their happiness using the Interdependent Happiness Scale (IHS). The relationship between IHS and Internet use (Japanese version of the Internet addiction test, JIAT), use of social networking services, as well as social function and sleep quality (Pittsburgh Sleep Quality Index, PSQI) were sought using multiple regression analyses.

**Results:**

Based on multiple regression analyses, the following factors related positively to IHS: female gender and the number of Twitter followers. Conversely, the following factors related negatively to IHS: poor sleep, high- PIU, and the number of times the subject skipped a whole day of school.

**Conclusions:**

It was shown that there was a significant negative correlation between Japanese youths’ happiness and PIU. Since epidemiological research on happiness that reflects the cultural background is still scarce, we believe future studies shall accumulate similar evidence in this regard.

## Background

With the growing influence of the Internet, our lifestyle has changed drastically within the last quarter century. The Internet penetration rate in Japan is 93.2%, which is the highest among Asian countries (e.g., Korea = 92.8%, Hong Kong = 87.5%, Singapore = 81.0%, Taiwan = 79.8%) [1]. The prevalence rate of Internet use is especially higher in younger generations than other age groups [2]. For young people in Japan, the Internet has become an indispensable communication tool [3]. In particular, Social Network Services (SNS) are one of the most popular ways for people to use the Internet in Japan. According to a survey conducted by the Ministry of Internal Affairs and Communications in 2015, the SNS utilization rate of Japanese in their teens and twenties, in terms of specific applications, was LINE (62.8%), Twitter (52.8) and Facebook (49.3). On the other hand, research and social criticism often emphasize the negative impact of Internet use. Typically, such studies investigate the relationship between mental health and Internet dependence, which is in many cases assessed by scales such as the Internet addiction test (IAT) [4], Chen Internet Addiction Scale (CIAS) [5], and others. For example, Kim et al. investigated the relationship between depression and Internet addiction in 1573 Korean adolescents using IAT, revealing that it was the Internet addiction group that showed a much higher degree of depression and suicidal ideation. Studies replicated the finding for depression [6, 7], but also for other psychiatric diseases; i.e. anxiety [8], obsessive compulsive disorder [9], personality disorder [10], attention deficit hyperactivity disorder (ADHD) [11], autism spectrum disorder (ASD) [12], and sleep disorder [13, 14] were found to be related to Internet dependence and/or problematic Internet use (PIU). Utilizing the data from the same population as we report in this paper, we also showed that multiple psychiatric symptoms, such as ADHD, poor sleep quality, and depressive state, were found to be related to PIU [15].

Besides research on psychiatric diseases related to PIU, a growing number of studies focus on the impact of the Internet on subjective well-being (SWB). There are already several review reports on SWB. For example, Çikrıkci conducted a meta-analysis on the impact of Internet usage on health satisfaction, happiness, and self-esteem as components of well-being [16]. Based on 23 studies that measured the relationship between Internet use and well-being, he concluded that PIU had a negative effect on well-being. Huang also meta-analyzed 40 studies that reported the relationship between Internet use and psychological well-being [17]. He defined depression, loneliness, self-esteem, and satisfaction with life as the components of psychological well-being and, unlike Çikrıkci, found that there was no relationship between Internet usage and psychological well-being. Although the results are inconsistent, the relationship between PIU and SWB has been a topic of interest lately. However, reports focusing on the Japanese population, the most digitally competent population in one of the most technologically advanced nations in the world, are very few to date.

There are multiple concepts and definitions surrounding SWB. For example, Diener et al. defines SWB as a person’s emotional and cognitive evaluations of their life, including what lay people call happiness, peace, fulfillment, and life satisfaction [18]. On the other hand, happiness is defined as “a global, subjective assessment of whether one is a happy or unhappy person” and is often considered one of the components of SWB [19]. Individuals with a high subjective feeling of well-being also have high mental health, quality of life, longevity, and physical health [20]. In this paper, we focused on a definition of happiness that includes individuals’ subjective feelings of happiness more than some other SWB concepts. In this thesis we focused on happiness wherein it is directly showing an individual’s subjective feelings of happiness within SWB composition concepts.

As a side note, how people recognize “happiness” greatly varies from country to country. Mesquita reported that lay people’s conceptions of happiness are best captured by exploring subjective experiences of happiness within a specific cultural context [21]. Uchida et al. compared the concept of happiness for American and Japanese subjects, and reported that while Americans tend to consider happiness to be a personal achievement, Japanese tend to think of happiness as social harmony [22]. Taking their argument seriously, we believe that in order to assess happiness in various countries, it is important to incorporate a culture’s concept of happiness.

In light of this cultural issue, Hitokoto et al. have developed a measure designed to capture happiness earned as a result of participating in and/or adapting to interdependent goals in life for Japanese subjects (i.e. the Interdependent Happiness Scale: IHS) [23]. IHS is a scale to measure the happiness of individuals who are relationally oriented, quiescent, and ordinary, and that more efficiently captures the meaning of happiness for Japanese people [23].

Previous studies have shown that people with PIU have more sleep problems [13, 14], and people with sleep problems spend more time watching television and using SNS [24]. There is also a report that showed that absenteeism increases due to poor sleep [25]. On the other hand, people with high SWB have fewer subjective sleep problems [26, 27]. This evidence seems to show that factors such as sleep quality or absenteeism could be potential mediators between PIU and happiness.

As mentioned above, from previous studies on the relationship between Internet use and happiness, there is little data for the Japanese population despite it being one of the world’s leading Internet users and there is a lack of consideration for differences in perception of happiness due to cultural differences. Therefore, we aimed to clarify the relationship between happiness and Internet use among Japanese university students whose Internet penetration rate is very high. Specifically, we investigated how their happiness could be explained by PIU, how they utilize SNS, as well as asking about their sleep functions and social functions (i.e. school performance, including the number of times a subject skipped a whole day of school and the number of times they arrived late for school).

## Methods

### Participants and consent

Five universities participated in this study. The number of students at these universities ranged from approximately 1000 to 30,000. The students majored in Sociology, Shinto, Medical technology, Physical therapy, and Nursing at universities. This study used a paper-based survey where respondents were asked to fill out self-report scales. Participants gave their consent by anonymously filling out the survey questionnaire after written and verbal explanation of the study from the researchers. This survey was conducted and collected in university classes and given only to students who were eager to participate in this research; however, an explanation of the study was given to all attendees in classes. Because the number of students who heard the explanation was not counted, we could not calculate the response rate of the survey. The target classes were arbitrarily chosen from classes listed in regular school schedules. The survey took place between January 2015 and November 2015. This study was approved by the Institutional Review Board of Keio University School of Medicine. In addition, each university obtained permission from its Institutional Review Board or equivalent committee. This study was conducted according to the principles of the Declaration of Helsinki.

### Outcome measures

Participants responded to questions about their age, gender, weekday and weekend Internet usage time, the number of Twitter follows and followers they have, the number of school days skipped, and the number of times they arrived late for school. They then completed the following three established measures.

### Happiness

We used the IHS by Hitokoto et al. [23] to assess happiness. IHS consists of nine items pertaining to an individual’s feeling of happiness, to which the subject responds with a 5-point Likert scale ranging from 1: strongly disagree to 5: strongly agree. The range of the score is 9–45, and the score indicates high happiness when the score is high (α = 0.82). This measure focuses on interpersonal harmony, ordinariness, and quiescence, and is validated amongst Japanese youth and adults (i.e. I believe that I and those around me are happy, I feel that I am being positively evaluated by others around me, I make significant others happy) [23].

### Problematic internet use

The Japanese version of the Internet addiction test (JIAT) (α = 0.91) [4] was used. This questionnaire consists of 20 questions about the subject’s Internet use. In this test, “Internet” includes all devices and applications used online, such as personal computers, mobile phones, smart phones, games, and so on. The subjects responded with a 5-point Likert scale (i.e., 1 = rarely, 2 = occasionally, 3 = frequently, 4 = often, 5 = always). The score range is 20–100, with higher scores indicating more severe addiction.

### Sleep quality

We used the Pittsburgh Sleep Quality Index (PSQI) to evaluate sleep [28]. PSQI is widely used as an evaluation of sleep disorders, and it asks nine questions about the state of the subject’s sleep in the past month. The questionnaire offers several measures of objective sleep quality, such as sleep latency, sleep duration, habitual sleep efficiency, sleep disturbance, use of sleeping medications, and daytime dysfunction. A higher score indicates worse sleep. The Japanese version of the PSQI, the PSQI-J, has good reliability (α = 0.77) [29].

### Internet usage time

The Internet usage time survey consists of a question regarding the subject’s Internet usage on weekdays and weekends. The question, which is used for both times of week, is: “How many hours a day do you use the Internet? This includes everything you use online such as a personal computer, cell phone, smartphone, game device, etc.” We asked for the average amount of time for each weekday and weekend day.

### Most-accessed internet application

We asked participants about their most-used Internet application. The choices were as follows: 1. SNS (Facebook, LINE, Twitter etc.), 2. Sending messages (email), 3. Making calls (including online calls), 4. Internet browsing (news, message boards, online shopping, reading online comics), 5. Online games, 6. Watching videos (YouTube etc.).

### Use of social networking services

This section consisted of three questions specific to SNS as follows: (1) “Do you use SNS (Facebook, mixi, Twitter, LINE)?” (Yes/No). (2) “How many individuals do you follow on Twitter? Please check your Twitter profile page and if you have multiple accounts, please indicate the total number of people”. (3) “How many followers do you have on Twitter? Please check your Twitter Profile screen and if you have more than one account please indicate the total number of people”.

### School performance (social functions)

We used two questions from the Program for International Student Assessment (PISA) test conducted by the Organization for Economic Co-operation and Development (OECD). The first question was “I skipped a whole day of school”, the second question was “I arrived late for school”. Answer options were: Never, Once or twice, Three or four times, Five or more times in the past 2 weeks [30].

### Statistical analysis

Distributions of all variables were inspected using histograms, q-q plots, and Shapiro-Wilks tests before conducting statistical analyses. Bonferroni Correction was carried out in order to take multiple comparisons into account.

We split the subjects into low and high groups using the median of the IHS score. Demographic characteristics, IAT, PSQI, Internet usage, PIU, and social functions were compared between the low- and high-IHS groups using t-test.

To empirically define the best fit model for IHS regression, we conducted a multiple regression analysis with a stepwise backward elimination. As for the predictor variables, we included sleep problems, PIU, Internet usage (weekday/weekend), number of Twitter follows/followers, number of school days skipped, number of times the subject arrived late for school, age, and sex. Variance Inflation Factor (VIF) was checked to evaluate if there was any multicollinearity among the predictor variables. All statistical analyses were performed using the SPSS software, version 23 (SPSS Inc., IBM, NC).

## Results

A total of 1336 subjects participated in the survey, but 78 were excluded due to missing data. Thus, the final sample comprised 1258 participants (male 544/1336, mean age ± SD = 19.3 ± 1.1). The mean IHS score (Mean ± SD) was 30.60 ± 6.53.

Table 1 shows the demographic characteristics and difference between high- and low-IHS groups regarding PIU, sleep states, Internet usage time, Twitter usage, and school performance. The high-IHS group had a significantly higher percentage of females, lower PIU tendency, higher sleep quality, shorter Internet use, and lower number of times skipping a whole day of school and arriving late for school, compared to the low-IHS group.

**Table 1:** The demographic characteristics and comparison of a low-IHS and high-IHS group following a median split, on PIU, sleep, internet usage time, twitter usage, and school performance

	Overall (*N* = 1258)		Low-IHS (*N* = 595)		High-IHS (*N* = 663)		t-value	*P* value*
	Mean	SD	Mean	SD	Mean	SD		
Total N (%)	1258 (100%)		595 (47.3%)		663 (52.7%)			
Sex (%)								
Male	544 (100%)		289 (53.1%)		255 (46.9%)		χ^2^ value 3.963	Male vs Female **< 0.001**
Female	714 (100%)		306 (42.9%)		408 (57.1%)			
Age	19.34	1.10	19.44	1.13	19.26	1.08	2.900	0.004
Happiness (IHS)	30.60	6.53	25.12	4.47	35.52	3.44	−45.893	**< 0.001**
IAT	37.87	12.59	40.62	13.43	35.40	11.24	7.438	**< 0.001**
PSQI global score	5.88	2.52	6.48	2.54	5.33	2.38	8.269	**< 0.001**
Subjective sleep quality	1.13	0.71	1.26	0.71	1.02	0.69	6.154	**< 0.001**
Sleep latency	0.99	0.92	1.15	0.96	0.84	0.85	6.165	**< 0.001**
Sleep duration	1.69	0.98	1.74	0.97	1.66	0.99	1.477	0.140
Habitual sleep efficiency	0.21	0.56	0.25	0.61	0.17	0.50	2.415	0.016
Sleep disturbance	0.68	0.50	0.75	0.49	0.62	0.51	4.914	**< 0.001**
Use of sleeping medications	0.04	0.32	0.05	0.33	0.03	0.30	0.874	0.382
Daytime dysfunction	1.13	0.71	1.28	0.87	1.00	0.89	5.529	**< 0.001**
Internet usage time/ min (Weekday)	205.79	147.70	215.69	159.08	196.95	136.25	2.224	0.026
Internet usage time/ min (Weekend)	277.63	183.86	296.74	197.60	260.63	169.04	3.434	**< 0.001**
Number of Twitter follows	218.18	393.53	215.35	456.81	220.70	327.14	−0.239	0.811
Number of Twitter followers	256.73	1354.17	280.50	1859.24	235.53	623.13	0.584	0.559
Number of times skipping a whole day of school	1.26	.551	1.33	0.64	1.19	0.45	4.206	**< 0.001**
Number of times arriving late for school	1.33	.655	1.41	0.76	1.25	0.54	4.295	**< 0.001**

*SD* standard deviation, *IHS* Interdependent Happiness Scale, *IAT* The Internet addiction test, *PSQI* The Pittsburgh Sleep Quality Index

*Bold text indicates a statistically significant difference after Bonferroni correction

Table 2 shows the percentage of Internet content that is used most frequently. The most frequently used Internet content was SNS among both males and females, followed by watching videos, Internet browsing, and online games. We also found a significant gender difference in the use of SNS and online games (*p* < 0.001).

**Table 2:** Percentage of internet content that is used most frequently in a day and differences between male and female

	Overall (%) (*N* = 1258)		Male (%) (*N* = 544)		Female (%) (*N* = 714)		χ^2^ value	*P* value*
	N	%	N	%	N	%		
SNS	645	51.3	216	39.7	429	60.1	51.320	< 0.001
Sending messages	10	0.8	5	0.9	5	0.7	0.188	1.000
Making calls	57	4.5	22	4.0	35	4.9	0.525	0.085
Internet browsing	169	13.4	69	12.7	100	14.0	0.464	0.017
Online games	153	12.2	103	18.9	50	7.0	41.141	< 0.001
Watching videos	189	15.0	112	20.7	77	10.8	23.245	0.011
Other	11	0.9	6	1.1	5	0.7	0.578	0.763
Unavailable	24	1.9	11	2.0	13	1.8	0.067	0.683

*SNS* social networking service

*Corrected for multiple testing with Bonferroni correction

Based on the multiple regression analyses (Table 3), the following factors related positively to IHS: female gender (β = 0.113. *p* < 0.001) and number of Twitter follows (β = 0.110, *p* < 0.001). The following factors related negatively to IHS: poor sleep (β = − 0.227, *p* < 0.001), high-PIU (β = − 0.220, *p* < 0.001), and number of school days skipped (β = − 0.083, *p* < 0.05) (*R*^*2*^ = 0.163). On the other hand, there were no significant associations between age, Internet usage time (weekday/weekend), number of Twitter followers, and number of times arriving late for school.

**Table 3:** Relationship between IHS and social functions

Response variables	Happiness (IHS)				
	Unstandardized Coefficients		Standardized Coefficients	t value	*P* value
	B	SE	β		
Sex	1.503	0.356	0.113	4.226	< 0.001
PSQI global score	−0.590	0.071	−0.227	−8.250	< 0.001
PIU (IAT)	− 0115	0.015	−0.220	−7.844	< 0.001
Number of Twitter follows	0.002	0.001	0.100	3.755	< 0.001
Number of times skipping a whole day of school	−0.994	0.324	−0.083	−3.068	< 0.01
R^2^	0.158				

*B* Beta, *SE* Standard error, *PSQI* The Pittsburgh Sleep Quality Index, *PIU* Problematic Internet use, *IAT* The Internet addiction test, *R*^*2*^ coefficient of determination. The predictor variables included sleep problems, PIU, Internet usage (weekday/weekend), number of Twitter follows/followers, number of school days skipped, number of times the subject arrived late for school, age, and sex

## Discussion

This study provides by far the largest epidemiological data investigating the relationship between happiness and Internet use among young Japanese people, an East Asian population with a high Internet penetration rate.

The results of this study revealed that the high happiness group had a significantly lower PIU tendency, a better sleep state, more number of Twitter follows and fewer days of school skipped compared to the low happiness group.

In response to the question about the content accessed online, SNS was the most frequent answer for males and females (51.3%). SNS is more frequently used by females than males (39.7% in males, 60.1% in females), while online games are more frequently used by males than females (male 18%, female 7.0%). The multiple regression analysis further showed that gender, sleep state, PIU, number of follows, and number of school days skipped predicted happiness.

Regarding the relationship between happiness and Internet dependence, Akin showed that there was a negative relationship between them [31], but few previous studies have focused on this topic [32, 33]. In previous studies on addiction and happiness, Long et al. reported that substance use reduced happiness [34]. Lanier et al. reported that university students’ drug use was negatively related to happiness [35]. Although with PIU the agent of dependence is different from substance dependence, we also found a negative correlation between happiness and PIU in this study population.

Previous studies have shown that people with high SWB have less subjective sleep problems [26, 27]. Although we assessed happiness, our data indicated similar findings; happiness was related to subjective sleep. We further found relationships between happiness and sleep quality, sleep latency, sleep efficiency, sleep disturbance, and daytime dysfunction which are subordinate items of PSQI. Because no significant association with happiness was found in sleep duration, high happiness may not be related to the length of sleep, but rather to the quality of sleep.

We found that the low happiness group used the Internet for longer periods of time compared to high happiness group. Sleep disorder caused by longer Internet use may be an underlying cause of this. Previous studies have shown that PIU is closely related to sleep disorders [13–15]. In addition, some researchers point out that social communication can be altered by Internet use, which may also be a contributing factor. Kraut suggested that Internet use may reduce real life communication and deepen one’s sense of isolation, thereby increasing depression [36]. It seems reasonable that one’s happiness can also be impaired by a decrease in communication due to Internet dependencies; however, this causality is yet to be determined.

Additionally, in this study, people with a low level of happiness tended to skip school and arrive late for school. Previous studies have shown that absenteeism increases due to poor sleep [25]. Lallukka followed adults in Finland for 7 years, revealing that both males and females have a high probability of absence when their sleeping time is shorter than 7.5 h or less (3–5 times for men, 2–3 times for women). The study also reported that absenteeism rates increase with stronger sleep disturbance (1.9 times for males, 1.4 times for females). Considering the fact that frequent absences also tend to lower scientific literacy [30], the lowered happiness and sleep deprivation may further deteriorate one’s willingness to attend school.

Because the sample population we used is very comfortable with technology, we especially paid attention to PIU in regards to Twitter, because Twitter is one of the most popular SNS in Japan. One of Twitter’s unique features is to “follow” and “be followed” by someone else. Following another person or account on Twitter is equivalent to subscribing to the posting (tweeting) of that person, and when one person subscribes to another person’s tweets, they are called a “follower”. A “mutual follow”, or the state in which two people both follow each other’s tweets, is known to lead to the stability of that relationship [37]. Among the general youth, one of their primary concerns regarding Twitter use is whether they will be followed back by someone whom they followed [38]. The number of followers one has can also be considered representative of the social popularity of that person [39]. These basic features of Twitter may be able to shed light on concerns about how one’s happiness and health are being affected by modern computer-mediated communication. “Follow” can be regarded as an active behavior to relate to others, while “to be followed” may reflect the established popularity of an individual within the Twitter society.

An interesting finding of our study was that happiness was predicted not by the number of “followers”, but by the number of “follows”. In the case of a public account, Twitter’s “follow” feature does not require the approval of those who are being followed. Therefore, a sense of high happiness may be related to the positive attitude and motivation that causes one to actively ask for connections with others.

The findings of this study need to be interpreted in the context of the following limitations. First, as this is a survey, we were not able to directly measure the subjects’ time spent using the Internet. Second, we were able to include five universities in Japan. However, four of those universities are located in urban areas and one is located in a rural area; therefore selection bias may exist. Moreover, since the study subject was limited to university students, we do not know whether the results of this research apply to all ages. Third, this study found a correlation between happiness, Internet usage, and healthy behavior. Some of the variables considered in this study, such as prolonged use of the Internet, poor sleep, and lack of social communication, may help us to understand the intricacies of happiness among modern youth. However, this study was cross-sectional, and no causal relations can be discovered through such a design. Fourth, in this study, we could not clearly distinguish and control the functions of the Internet content accessed by the study subjects. For example, we treated sending messages, making calls, Internet browsing, online games, and watching videos as independent content separate from SNS, but since all those features also exist as functions of SNS, there may be overlap. Fifth, we could not calculate the response rate of the survey because the number of students who heard the explanation was not counted. Finally, we focused solely on the amount of followers or number of times followed; this does not necessarily reflect current activities online, as a student might have registered with Twitter in the past and interacted with it on a regular basis for several years, but not have been as active at the time of the study.

## Conclusions

In summary, we showed that there was a significant negative correlation between Japanese youths’ happiness and PIU. Additionally, happiness was also positively related to number of Twitter follows and female gender, and negatively related to poor sleep and number of school days skipped. Since epidemiological research on happiness is still scarce, we believe future studies shall accumulate similar evidence in this regard. For example, a prospective study that examines the relationship between happiness and Internet use by closely monitoring user content, or a study that identifies factors that contribute to one’s happiness by collecting participants’ virtual and actual daily life logs, may be valuable.

## Data Availability

The datasets used and/or analysed during the current study are available from the corresponding author on reasonable request.

## Abbreviations

ADHD: Attention deficit hyperactivity disorder
CIAS: Chen Internet Addiction Scale
IAT: The Internet addiction test
IHS: The Interdependent Happiness Scale
JIAT: Japanese version of the Internet addiction test
OECD: The Organization for Economic Co-operation and Development
PIU: Problematic Internet use
PSQI: Pittsburgh Sleep Quality Index
SNS: Social Network Services
SWB: Subjective well-being
VIF: Variance Inflation Factor
